# C1q/TNF-Related Protein 9 Inhibits Coxsackievirus B3-Induced Injury in Cardiomyocytes through NF-*κ*B and TGF-*β*1/Smad2/3 by Modulating THBS1

**DOI:** 10.1155/2020/2540687

**Published:** 2020-12-19

**Authors:** Kebei Liu, Juan Wang, Xinru Gao, Wei Ren

**Affiliations:** ^1^Department of Internal Medicine, Xi'an Children's Hospital, Xi'an, Shaanxi 710003, China; ^2^Department of Clinical Laboratory, Xi'an Children's Hospital, Xi'an, Shaanxi 710003, China; ^3^Department of Medical Ultrasound Center, The Northwest Women's and Children's Hospital, Xi'an, Shaanxi 710003, China

## Abstract

C1q/TNF-related protein 9 (CTRP9) is implicated in diverse cardiovascular diseases, but its role in viral myocarditis (VMC) is not well explored. This study is aimed at investigating the role and potential mechanism of CTRP9 in VMC. Herein, we found that the peripheral blood collected from children with VMC had lower CTRP9 levels than that from children who had recovered from VMC. H9c2 cardiomyocytes treated with coxsackievirus B3 (CVB3) were applied to establish a VMC model *in vitro*, and the expression of CTRP9 was significantly decreased in CVB3-induced H9c2 cells. The overexpression of CTRP9 attenuated CVB3-induced apoptosis, inflammation, and fibrosis reactions in H9c2 cells by promoting cell proliferation, reducing the cell apoptosis rate, and inhibiting inflammatory cytokine levels and fibrosis-related gene expression. Moreover, we found that thrombospondin 1 (THBS1) levels were increased in children with VMC, and CTRP9 negatively regulated THBS1 expression by interacting with THBS1. The downregulation of THBS1 inhibited CVB3-induced apoptosis, inflammation, and fibrosis in H9c2 cells. In addition, our mechanistic investigation indicated that the overexpression of THBS1 impaired the inhibitory effect of CTRP9 on CVB3-induced H9c2 cells. The results further revealed that the CVB3-induced NF-*κ*B and TGF-*β*1/Smad2/3 signaling pathways of H9c2 cells were blocked by CTRP9 yet activated by THBS1. In conclusion, CTRP9 protected H9c2 cells from CVB3-induced injury via the NF-*κ*B and TGF-*β*1/Smad2/3 signaling pathways by modulating THBS1.

## 1. Introduction

Viral myocarditis (VMC) is a common cardiovascular disease in the clinic [1]. It is localized or diffuse myocarditis caused by viral infection, which can cause localized or extensive myocardial interstitial inflammatory infiltration and myocardial cell necrosis or apoptosis, eventually leading to irreversible cardiac dysfunction [2]. In recent years, the incidence of VMC in our country has gradually increased [3, 4]. In addition, the incidence of VMC in children is higher than in adults, which is an important cause of sudden childhood death [5]. Therefore, clarifying the pathogenesis of VMC in children is of great significance for breakthroughs in clinical treatment.

Coxsackievirus B3 (CVB3) is the most common pathogenic virus causing VMC [2, 3, 6]. Studies have shown that CVB3 can induce the NF-*κ*B of cardiomyocytes to enter the nucleus from the cytoplasm, leading to a significant increase in the secretion of inflammatory factors and others, thereby regulating inflammation and immune responses and promoting the development of VMC [7, 8]. Transforming growth factor- (TGF-) *β*1 is one of the most important precipitating factors in tissue fibrosis [9]. It affects the expression of extracellular matrix proteins by activating the downstream Smad protein family [9, 10]. The sustained high expression of TGF-*β*1 is closely related to the increase in collagen deposition in the chronic phase of VMC [11]. Studies have reported that CVB3 induces excessive activation of the TGF-*β*1/Smad signaling pathway to aggravate the pathological process of VMC myocardial fibrosis [12]. Moreover, downregulating the expression of myocardial TGF-*β*1 can reduce viral myocardial inflammatory damage [11–13].

C1q/TNF-related protein 9 (CTRP9) belongs to the CTRP superfamily, and its spherical domain (active region) has the highest homology with adiponectin (APN) [14, 15]. CTRP9 is a type of adipokine that plays an important protective role in regulating metabolism, inhibiting liver steatosis, and improving insulin resistance [16]. Recent studies have found that CTRP9 is closely related to various cardiovascular diseases [17]. Serum CTRP9 levels are significantly increased in patients with type 2 diabetes and coronary heart disease, and they are closely related to inflammation levels [18]. The overexpression of CTRP9 can inhibit myocardial fibrosis in diabetic rats [19]. In addition, other studies have found that CTRP9 reduces myocardial ischemia/reperfusion injury by inhibiting cardiomyocyte apoptosis [15, 20]. What is more, recent studies have indicated that human-derived recombinant CTRP9 can attenuate isoproterenol-induced myocardial injury in mice [14]. These findings suggest that CTRP9 has a protective effect on the heart, but its specific mechanism of action in VMC is still unclear.

The thrombospondin (THBS) family is a class of calcium-related glycoproteins with multiple domains, which are mainly located on the surface of cell membranes [21]. Due to their special trimeric structure, THBS are widely involved in many biological processes such as angiogenesis, cell movement and apoptosis, cytoskeletal composition, and extracellular matrix (ECM) reaction, through interactions with various target proteins [22, 23]. Moreover, previous papers have shown that the inhibition of THBS1 ameliorates fibrotic remodeling processes in aging cardiomyocytes, and the loss of THBS1 regulates the fibroblast phenotype and matrix remodeling to aggravate chamber dilation [24, 25]. However, its role in the development of VMC remains unclear.

This study attempted to demonstrate the role of CTRP9 in a CVB3-induced VMC cell model. Interestingly, it is reported that APN can directly interact with THBS1 [26], but whether CTRP9 interacts with this protein remains unknown. In addition, THBS1 modulates the inflammatory response via NF-*κ*B signaling, and it can also act as an activator of TGF-*β*1 signaling [27, 28]. Therefore, our work investigated the relationship between CTRP9, THBS1, and the downstream NF-*κ*B and TGF-*β*1/Smad2/3 pathways, to determine whether the THBS1 and NF-*κ*B and TGF-*β*1/Smad2/3 pathways are involved in CTRP9-mediated protective effects on VMC. This finding might provide a potential strategy for VMC treatment.

## 2. Materials and Methods

### 2.1. Patients

A total of 35 cases of VMC children aged 9 months to 11 years old who were admitted to our hospital from July 2015 to March 2019 were included in this study. Since 7 patients with other diseases were excluded, only 28 cases were included in the VMC group. All children with VMC received symptomatic treatment, myocardial nutrition and antiviral treatment, and the protection of important organ functions. After the treatment, 24 cases (aged 9 months to 11 years old) finally recovered and were included in the control group; they were followed up regularly after rehabilitation. When fasting early in the morning, peripheral blood was collected from all sick and control children and stored in EDTA anticoagulation tubes at -20°C. All procedures were approved by the Medical Ethics Committee of Xi'an Children's Hospital. All patients or families provided written informed consent.

### 2.2. Cell Culture and Treatment

H9c2 cells, obtained from the American Type Culture Collection, were plated at a density of 5 × 10^5^ cells/well into 6-well plates. The cells were cultured in Dulbecco's modified Eagle's medium (Invitrogen Life Technologies, Carlsbad, CA, USA) supplemented with 10% fetal calf serum (Invitrogen Life Technologies, Carlsbad, CA, USA) in 95% air and 5% CO_2_ at 37°C for incubation for 24 h. Then, H9c2 cells were infected with 2 × 10^4^ pfu/mL CVB3 (Centre for Endemic Disease Control, Beijing, China) for 48 h.

### 2.3. Transfection

Full-length rat CTRP9 (PubMed No. NM_001191891.1) and full-length rat THBS1 (PubMed No. NM_001013062.1) were synthesized by Sangon Biotech Co., Ltd. (Shanghai, China) and inserted into recombinant adenovirus vectors, using the same construction protocol as the previous study [29]. The small interfering RNA against THBS1 (si-THBS1) was synthesized by GenePharma Co., Ltd. (Shanghai, China). The vectors were transfected into H9c2 cells by using Lipofectamine 3000 (Invitrogen, Carlsbad, CA, USA). After transfection for 48 h, the transfection efficiency was more than 90%, which proved that the virus transfection was successful. Then, H9c2 cells were treated with CVB3 (2 × 10^4^ pfu/mL) for 48 h.

### 2.4. Quantitative Real-Time PCR (qRT-PCR)

The total RNA from serum samples or H9c2 cells was extracted using the TRIzol reagent (Invitrogen, Shanghai, China) according to the manufacturer's protocol. The isolated RNA (2 *μ*g) was reverse-transcribed to cDNA using a SuperScript® III First-Strand Synthesis System (Invitrogen, Shanghai, China). qRT-PCR was performed using the Real-Time Quantitative PCR SYBR Green Kit (Takara, Tokyo, Japan). The relative mRNA levels were normalized to GAPDH and calculated using the 2^−*ΔΔ*Ct^ method.

### 2.5. Enzyme-Linked Immunosorbent Assay (ELISA)

After centrifugation, serum was collected from the peripheral blood of the patients, and the supernatant culture medium of H9c2 cells was also collected for analysis. The concentrations of CTRP9, THBS1, TNF-*α*, IL-6, and IL-1*β* were detected using ELISA kits (R&D Systems, Minneapolis, USA) according to the manufacturer's instructions.

### 2.6. Cell Proliferation Assay

H9c2 cell viability was detected using the Cell Counting Kit-8 (CCK-8, Dojindo, Japan). Briefly, cells were seeded at a density of 2 × 10^3^ cells per well in 96-well plates. After being cultured for 48 h, cells were incubated with 10 *μ*L CCK-8 solution per well at room temperature for 2 h, and the optical density (OD) absorbed by the tested objects was measured using a microplate reader (Bio-Rad, CA, USA) at 450 nm.

### 2.7. Cell Apoptosis Assay

The apoptosis rate of H9c2 cells was analyzed with an Annexin V-fluorescein isothiocyanate (FITC) conjugate combined with propidium iodide (PI) assay (Dojindo, Japan) and evaluated by fluorescence-activated cell sorting (BD Biosciences, San Diego, USA).

### 2.8. Western Blot and Coimmunoprecipitation (Co-IP)

After treatment, H9c2 cells were collected, and the total protein was extracted after being lysed with the RIPA lysis buffer (Beyotime Biotechnology, Inc., Shanghai, China). Part of the total protein samples quantified by the BCA method was electrophoresed on an SDS-PAGE gel, and the electrophoresis was completed after the target protein was separated. After transferring the proteins to a PVDF membrane, membranes were blocked by 5% nonfat milk for 2 h. Next, the primary antibody solution was added and samples were incubated for 2 h at room temperature; then, the secondary antibody was added for 1.5 h at room temperature. After Pierce ECL Western Blotting Substrate (Thermo Fisher Scientific, Massachusetts, USA) treatment, the ImageJ software (National Institutes of Health, Maryland, USA) was used to analyze the relative expression level of the target protein with GAPDH as an internal control. For the Co-IP assay, after the extracted protein was quantified by the BCA method, the relevant antibodies and protein A/G beads (Thermo Fisher Scientific, Massachusetts, USA) were added to the protein lysates and then incubated at 4°C overnight. Afterwards, the immunocomplex samples were washed with the precold IP lysis buffer three times, and the supernatant was collected for Western blot.

### 2.9. Statistical Data Analysis

Experiments were performed in triplicate with at least three repetitions. Statistical analysis was conducted using SPSS version 19.0 statistical software and GraphPad Prism 5.0 software. Pearson's *χ*^2^ test was used to examine the correlation between the CTRP9 and THBS1 levels in the serum of children with viral myocarditis (VMC). Student's *t*-test and one-way ANOVA were used to compare the significance of differences between groups. The data are expressed as mean ± SD, and all statistical significances were set at *P* < 0.05.

## 3. Results

### 3.1. CTRP9 Was Poorly Expressed in Viral Myocarditis

**Figure 1:**
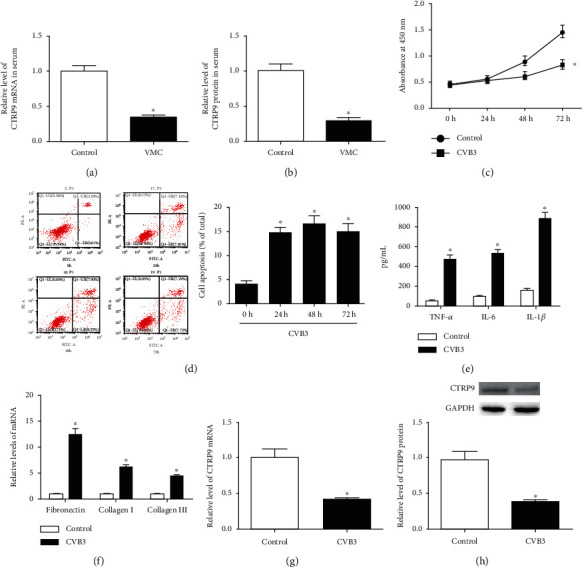
CTRP9 was poorly expressed in viral myocarditis. (a, b) Children with VMC were included in the VMC group (*n* = 28), and those who have recovered from VMC were included in the control group (*n* = 24). qRT-PCR was used to measure the expression of CTRP9 mRNA in serum, while ELISA was used to determine CTRP9 protein contents in serum. (c) H9c2 cells were infected with CVB3 (2 × 10^4^ pfu/mL) for 24 h, 48 h, or 72 h; H9c2 cardiomyocytes that didn't infect with CVB3 were. Cell proliferation was measured by CCK-8 assay. (d) Apoptosis rate was detected by flow cytometry. (e) After treated with CVB3 for 48 h, the secretion levels of inflammatory cytokines, including TNF-*α*, IL-6, and IL-1*β*, in the medium of H9c2 cells were measured by ELISA; nontreated H9c2 cells performed as the control. (f) The mRNA expression of fibrosis-related genes, including fibronectin, collagen I, and collagen III, was detected by qRT-PCR. (g, h) The expression of CTRP9 mRNA and protein was measured by qRT-PCR and Western blot, respectively. ^∗^*P* < 0.05 vs. the control group. All the experiments were repeated at least three times. VMC: viral myocarditis; CVB3: coxsackievirus B3; CCK-8: Cell Counting Kit-8; TNF-*α*: tumor necrosis factor *α*; IL-6: interleukin 6; IL-1*β*: interleukin 1*β*; ELISA: enzyme-linked immunosorbent assay.

To clarify the role of CTRP9 in pediatric viral myocarditis (VMC), the expression of CTRP9 mRNA and protein in the serum of children with VMC was detected by qRT-PCR and ELISA. There was no significant difference in serum CTRP9 mRNA expression between the different age groups in VMC children, suggesting that growth does not affect CTRP9 levels in children (SFig. 1). Also, our data showed that children with VMC have lower CTRP9 levels than children who have recovered from VMC, suggesting that CTRP9 might play a role in VMC (Figures 1(a) and 1(b), *P* < 0.05). In addition, we established the VMC cell model by H9c2 cardiomyocytes infected with CVB3. As shown in Figures 1(c) and 1(d), CVB3 infection significantly inhibited cell proliferation, while it enhanced the cell apoptosis of H9c2 cells (*P* < 0.05). The ELISA data indicate that CVB3 elevated the levels of the inflammatory cytokines TNF-*α*, IL-6, and IL-1*β* in H9c2 cells, and the qRT-PCR data further revealed that CVB3 promoted the mRNA expression of the fibrosis-related genes fibronectin, collagen I, and collagen III in H9c2 cells, suggesting the successful establishment of CVB3-induced myocarditis (Figures 1(e) and 1(f), *P* < 0.05). Then, the expression of CTRP9 in CVB3-induced H9c2 cells was determined, and the results demonstrated that CTRP9 was low expressed in H9c2 cells after CVB3 infection (Figures 1(g) and 1(h), *P* < 0.05).

### 3.2. CTRP9 Protected H9c2 Cells against CVB3-Induced Apoptosis, Inflammation, and Fibrosis

**Figure 2:**
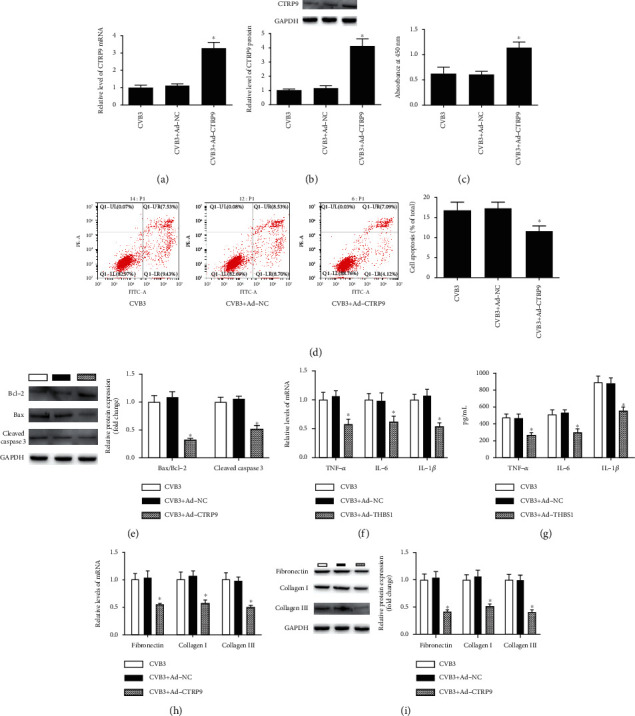
CTRP9 protected H9c2 cells against CVB3-induced apoptosis, inflammation, and fibrosis. (a, b) H9c2 cells were transfected with recombinant adenovirus vectors containing full-length rat CTRP9 (Ad-CTRP9) or its control vectors (Ad-NC) for 48 h before infected with CVB3. Then, the expression of CTRP9 mRNA and protein of H9c2 cells was measured. (c) The cell viability and (d) the apoptosis rate were detected after Ad-CTRP9 transfection. (e, f) The protein expression of apoptosis-related factors, including Bcl-2, Bax, and cleaved caspase 3, was measured after Ad-CTRP9 transfection. (f, g) The mRNA expression and secretion levels of proinflammatory factors TNF-*α*, IL-6, and IL-1*β* were measured after Ad-CTRP9 transfection. (h, i) The mRNA and protein expression of pro-fibrosis-related genes fibronectin, collagen I, and collagen III was detected after Ad-CTRP9 transfection. ^∗^*P* < 0.05 vs. the CVB3 group. *n* = 6 in each group. Bcl-2: B-cell lymphoma-2; Bax: B-cell lymphoma-2-associated X; TNF-*α*: tumor necrosis factor *α*; IL-6: interleukin 6; IL-1*β*: interleukin 1*β*.

To assess the effect of CTRP9 on CVB3-induced apoptosis, recombinant adenovirus vectors containing full-length rat CTRP9 (Ad-CTRP9) were transfected into H9c2 cells before CVB3 infection. The data of qRT-PCR and Western blot indicated that Ad-CTRP9 conspicuously improved the expression of CTRP9 mRNA and protein in CVB3-induced H9c2 cells (Figures 2(a) and 2(b), *P* < 0.05). The cell viability of CVB3-induced H9c2 cells was significantly increased, while the cell apoptosis rate was significantly decreased after Ad-CTRP9 transfection (Figures 2(c) and 2(d), *P* < 0.05). Moreover, the protein expression of Bax/Bcl-2 and cleaved caspase 3 of CVB3-induced H9c2 cells also showed a decrease following Ad-CTRP9 transfection (Figures 2(e) and 2(f), *P* < 0.05). The effect of CTRP9 on CVB3-induced inflammation and fibrosis in H9c2 cells was further explored. Our data showed that Ad-CTRP9 prominently attenuated the increase in CVB3 on mRNA expression and secretion levels of TNF-*α*, IL-6, and IL-1*β* in H9c2 cells (Figures 2(f) and 2(g), *P* < 0.05). With respect to fibrosis, the mRNA and protein expression of fibronectin, collagen I, and collagen III in CVB3-induced H9c2 cells was prominently reduced by Ad-CTRP9 transfection (Figures 2(h) and 2(i), *P* < 0.05). These data suggest that CTRP9 protected H9c2 cells from CVB3-induced apoptosis, inflammation, and fibrosis.

### 3.3. CTRP9 Negatively Regulated THBS1 Expression in CVB3-Infected H9c2 Cells

**Figure 3:**
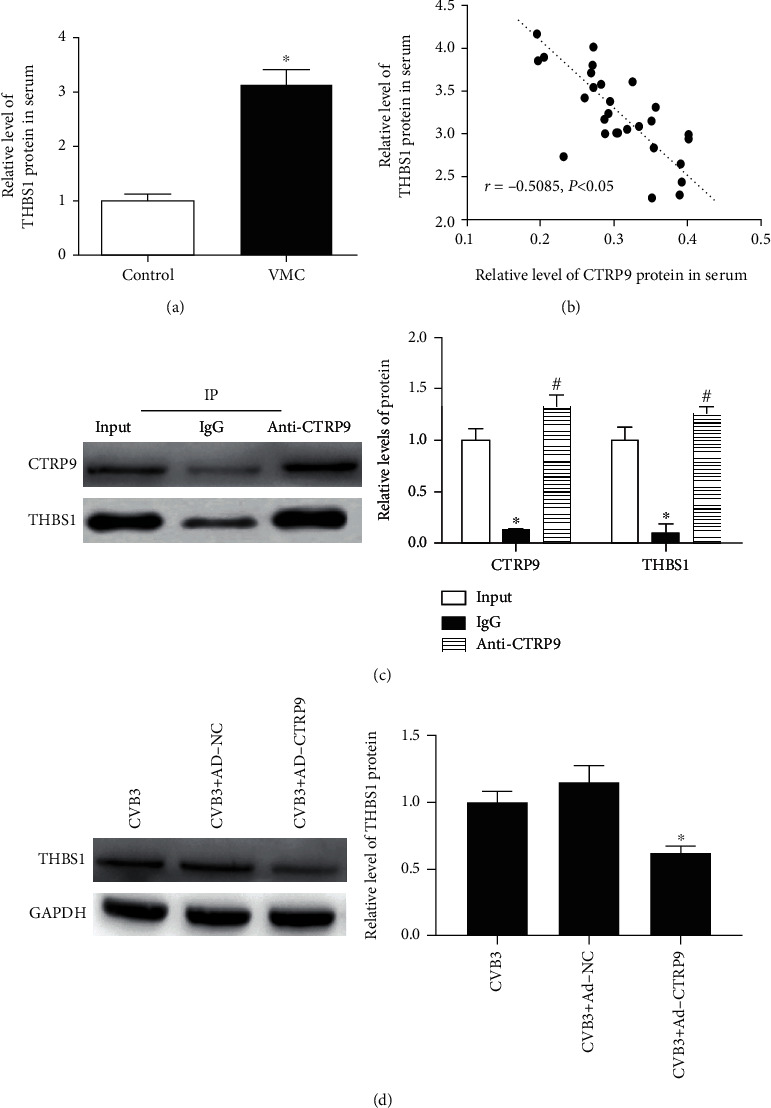
CTRP9 negatively regulated THBS1 expression in CVB3-infected H9c2 cells. (a) ELISA was used to determine THBS1 protein contents in the serum of the control and VMC groups. ^∗^*P* < 0.05 vs. the control group. *n* = 6 in each group. (b) The correlations among CTRP9 protein contents and THBS1 protein contents in the VMC group were determined by Pearson's correlation analysis (*r* = −0.5085, *P* < 0.05). (c) The interaction between CTRP9 protein and THBS1 protein was determined by coimmunoprecipitation (Co-IP) in H9c2 cells, with IgG as a negative control. ^∗^*P* < 0.05 vs. input. ^#^*P* < 0.05 vs. IgG. *n* = 6 in each group. (d) After Ad-CTRP9 transfection, the protein expression of THBS1 of H9c2 cells was detected by Western blot. ^∗^*P* < 0.05 vs. the CVB3 group. *n* = 6 in each group.

**Figure 4:**
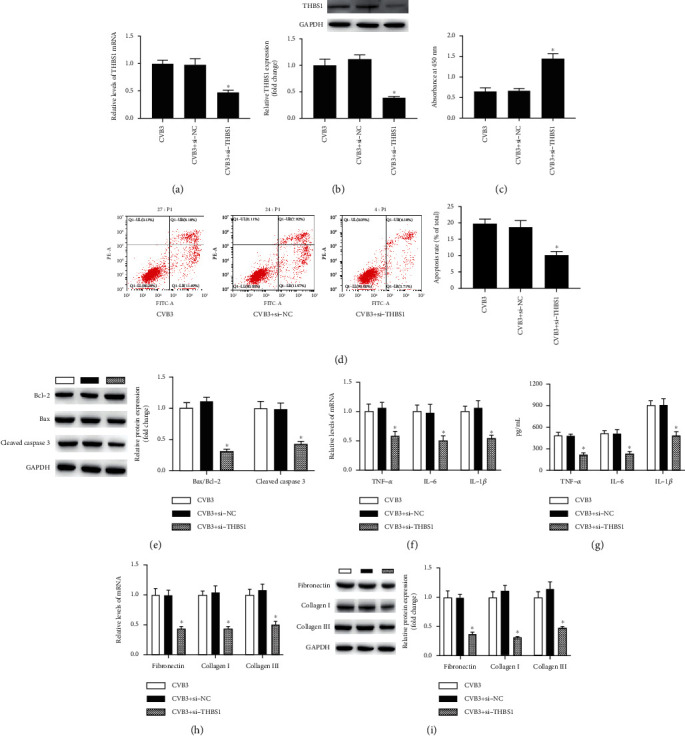
Inhibition of THBS1 protected H9c2 cells against CVB3-induced apoptosis, inflammation, and fibrosis. (a, b) H9c2 cells were transfected with the small interfering RNAs against THBS1 (si-THBS1) or its control vectors (si-NC) for 48 h before infected with CVB3. Then, the expression of THBS1 mRNA and protein of H9c2 cells was detected. (c) The cell viability and (d) the apoptosis rate were detected after si-THBS1 transfection. (e, f) The protein expression of Bcl-2, Bax, and cleaved caspase 3 of CVB3-induced H9c2 cells was determined after si-THBS1 transfection. (f, g) The mRNA expression and the secretion levels of TNF-*α*, IL-6, and IL-1*β* were measured after si-THBS1 transfection. (h, i) After si-THBS1 transfection, the mRNA and protein expression of fibronectin, collagen I, and collagen III was detected. ^∗^*P* < 0.05 vs. the CVB3 group. *n* = 6 in each group.

The ELISA data showed that THBS1 protein levels in the serum of children with VMC were higher than those of children who had recovered from VMC (Figure 3(a), *P* < 0.05). Interestingly, Pearson's correlation scatterplot showed that CTRP9 levels were negatively correlated with THBS1 levels in children with VMC (Figure 3(b), *P* < 0.05). Furthermore, the Co-IP assay was applied to determine whether CTRP9 interacts with the THBS1 protein. As expected, Western blot successfully identified THBS1 protein in anti-CTRP9 rather than IgG pretreated immunocomplex samples, implying that CTRP9 interacted with THBS1 (Figure 3(c), *P* < 0.05). In addition, the data in Figure 4(d) indicate that THBS1 protein expression was negatively regulated by CTRP9 in CVB3-infected H9c2 cells (Figure 3(d), *P* < 0.05).

### 3.4. Inhibition of THBS1 Protected H9c2 Cells against CVB3-Induced Apoptosis, Inflammation, and Fibrosis

To investigate whether the inhibition of THBS1 plays a role in the pathogenesis of VMC, we inhibited THBS1 expression in CVB3-induced H9c2 cells through its small interfering RNA (si-THBS1). As indicated in Figures 4(a) and 4(b), si-THBS1 significantly reduced the expression of THBS1 mRNA and protein in CVB3-induced H9c2 cells (*P* < 0.05). Then, functional experiments revealed that si-THBS1 increased cell viability while decreasing the apoptosis rate and the expression of proapoptotic proteins in H9c2 cells after CVB3 stimulation (Figures 4(c)–4(e), *P* < 0.05). Further data showed that CVB3-induced proinflammatory factors and pro-fibrosis-related gene expression were inhibited after the transfection of si-THBS1 in H9c2 cells (Figures 4(h) and 4(i), *P* < 0.05). Therefore, the inhibition of THBS1 negatively regulated CVB3-induced apoptosis, inflammation, and fibrosis in H9c2 cells.

### 3.5. THBS1 Attenuated the Effect of CTRP9 on CVB3-Induced H9c2 Cells

**Figure 5:**
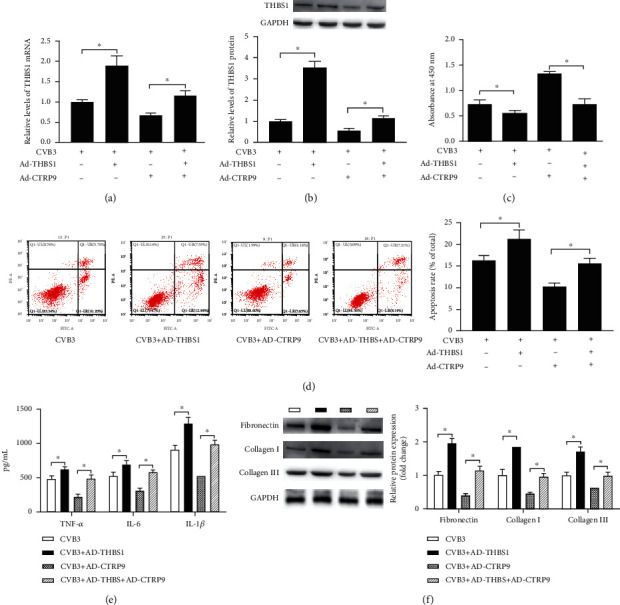
THBS1 attenuated the effect of CTRP9 on CVB3-induced H9c2 cells. (a, b) After transfected with recombinant adenovirus vectors containing full-length rat THBS1 (Ad-THBS1) or/and Ad-CTRP9, the mRNA and protein expression of THBS1 in CVB3-infected H9c2 cells was detected. (c) The cell viability, (d) apoptosis rate, (e) inflammatory cytokine levels, and (f) fibrosis-related gene expression were measured after Ad-THBS1 or/and Ad-CTRP9 transfection. ^∗^*P* < 0.05. *n* = 6 in each group.

Subsequently, the present study investigated the association between THBS1 and CTRP9 in CVB3-induced H9c2 cells. The recombinant adenovirus vectors containing full-length rat THBS1 (Ad-THBS1) were shown to significantly increase THBS1 mRNA and protein expression in CVB3-induced H9c2 cells (Figures 5(a) and 5(b), *P* < 0.05). Afterwards, THBS1 showed a promotional role in CVB3-induced apoptosis, inflammation, and fibrosis by inhibiting cell viability, enhancing the cell apoptosis rate, inflammatory cytokine levels, and fibrosis-related gene expression (Figures 5(c)–5(f), *P* < 0.05). Moreover, THBS1 diminished the inhibitory influence of CTRP9 on CVB3-induced apoptosis, inflammation, and fibrosis, suggesting that CTRP9 played a protective role in CVB3-infected H9c2 cells by interacting with THBS1.

### 3.6. CTRP9 Blocked NF-*κ*B and TGF-*β*1/Smad2/3 Signaling Pathways via Modulating THBS1

**Figure 6:**
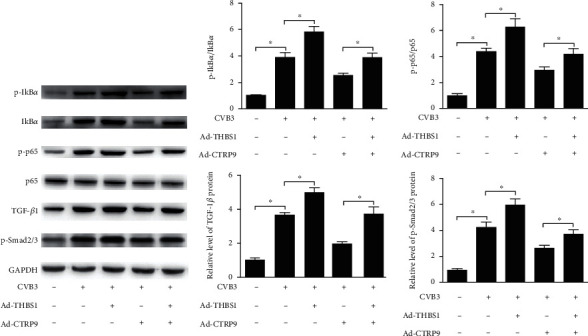
CTRP9 blocked NF-*κ*B and TGF-*β*1/Smad2/3 signaling pathways via modulating THBS1. After transfected with Ad-CTRP9 or/and Ad-THBS1, the protein expression of NF-*κ*B and TGF-*β*1/Smad2/3 signaling pathway-related factors, including p-I*κ*B*α*, I*κ*B*α*, p-p65, p65, TGF-*β*1, and p-Smad2/3, in CVB3-infected H9c2 cells was determined by Western blot. ^∗^*P* < 0.05. *n* = 6 in each group.

It has been reported that the nuclear factor-*κ*B (NF-*κ*B) and transforming growth factor-*β*1 (TGF-*β*1)/Smad2/3 signaling pathways are regulated by THBS1 or CTRP9 in various cardiology diseases [19, 28, 30]; thus, we hypothesized that CTRP9 regulated these signaling pathways via modulating THBS1 in CVB3-infected H9c2 cells. The Western blot data determined that Ad-CTRP9 blocked the activation of the NF-*κ*B and TGF-*β*1/Smad2/3 signaling pathways by suppressing the protein expression of p-I*κ*B*α*/I*κ*B*α*, p-p65/p65, TGF-*β*1, and p-Smad2/3 in CVB3-infected H9c2 cells, while THBS1 impaired this effect (Figure 6, *P* < 0.05).

## 4. Discussion

VMC is a systemic inflammatory cardiovascular disease, with a high mortality rate among adolescents [2]. Since CVB3-induced VMC is very similar to the disease characteristics of human myocarditis, it has become a widely used model of VMC [3, 7]. Consistent with previous observations in VMC [31–34], our study also indicated that CVB3 infection can significantly promote apoptosis, inflammation, and fibrosis reactions, as demonstrated by the increased expression of the apoptosis-related genes Bax and cleaved caspase 3, increased levels of TNF-*α*, IL-6, and IL-1*β*, and increased levels of the fibrosis-related genes fibronectin, collagen I, and collagen III. In addition, we found that CTRP9 expression was downregulated in children with VMC and CVB3-induced H9c2 cells.

The involvement of CTRP9 was frequently observed in ischemic cardiac diseases and may play an important protective role in the cardiovascular system [15, 20]. It has been reported that CTRP9 gene knockout mice show increased areas of myocardial infarction and promote cardiac dysfunction after myocardial ischemia-reperfusion (IR) injury [15, 35]. In addition, CTRP9 gene knockout mice can aggravate left ventricular systolic and diastolic dysfunction, myocardial cell apoptosis, and inflammation in the ischemic heart [20], while the overexpression of CTRP9 can reduce the area of myocardial infarction after myocardial IR injury in normal or diabetic mice, and supplementing wild-type mice with the CTRP9 protein can improve cardiac function, apoptosis, and fibrosis after myocardial infarction [36, 37]. Furthermore, CTRP9 can inhibit hypoxia-induced apoptosis and LPS-induced inflammation by activating AMPK or cAMP in cardiomyocytes [17]. In this study, we found that the overexpression of CTRP9 protected H9c2 cells against CVB3-induced apoptosis, inflammation, and fibrosis by interacting with THBS1.

THBS1 is a matricellular protein, which was initially identified in the extracellular matrix (ECM) [38]. Normally, THBS1 shows a low expression within ECM, but in the tissue injury or changed cellular environment, THBS1 expression is dramatically elevated. Increased THBS1 levels can stimulate the expression of the components of ECM, including fibronectin and collagens such as types I and III [24, 39]. It has been well demonstrated that the excessive accumulation of ECM components is responsible for the development of cardiac fibrosis; thus, THBS1 plays a key role in human fibrosis diseases [24, 38]. Moreover, THBS1 contributes to vascular inflammation in the abdominal aortic aneurysm through activation of TGF-*β*1 [40, 41], and THBS1 mediates endothelial cell apoptosis by activating caspase [42]. Our findings indicated that THBS1 is involved in CVB3-induced apoptosis, inflammation, and fibrosis reactions in H9c2 cells. It has been reported that the spherical domain (active region) of CTRP9 has the highest homology with adiponectin (APN) [14, 15], and APN can directly interact with THBS1 [26]. Our Co-IP results showed that CTRP9 can bond with THBS1, and CTRP9 repressed CVB3-induced injury in H9c2 cells via interacting with THBS1.

TGF-*β*1 is a versatile polypeptide that plays an important role in regulating cell growth, differentiation, and the repair of various tissues [9]. In addition, TGF-*β*1 can participate in the regulation of myocardial fibroblast proliferation, transformation, migration, and ECM production via the TGF-*β*/Smad signaling pathway [9, 10]. A number of studies have shown that inhibition of the TGF-*β*1/Smad2/3 signaling pathway relieves the cardiomyocyte apoptosis and fibrosis reactions in myocarditis [43, 44]. In addition, the TGF-*β*1/Smad2/3 signaling pathway has a proinflammatory role in multiple injured tissues and can mediate mitochondrial apoptosis via regulating Bcl-2 expression [45, 46]. It is now clear that THBS1 is one of the most important physiological activators of TGF-*β*1, and research has shown that the THBS1-activated TGF-*β*1/Smad2/3 signaling pathway may play an important role in the development of myocardial interstitial fibrosis in diabetic cardiomyopathy [47]. NF-*κ*B is a protein molecule with multiphase regulation, plays an important role in inflammatory response and immune regulation, and is regarded as the central regulator of cardiomyopathy [8]. Besides, the inactivation of NF-*κ*B has been shown to alleviate the injuries of CVB3-induced VMC *in vivo* and *in vitro* [48, 49]. THBS1 can regulate inflammatory cytokine secretion and angiogenesis by activating NF-*κ*B, and CTRP9 attenuates atrial inflammation and fibrosis by suppressing the NF-*κ*B and Smad2/3 signaling pathways [19, 28, 30]. The present study shows that CTRP9 attenuated CVB3-induced injury via the NF-*κ*B and TGF-*β*1/Smad2/3 signaling pathways by negatively modulating THBS1 expression in H9c2 cells.

## 5. Conclusions

The present study found that CTRP9 was downregulated and THBS1 was upregulated in children with VMC and H9c2 cells after CVB3 stimulation. Our research further demonstrated that CTRP9 interacted with THBS1 to alleviate CVB3-induced injury by blocking the NF-*κ*B and TGF-*β*1/Smad2/3 signaling pathways, which could provide an effective strategy for VMC treatment. There are some limitations to this study. Since mammalian cardiomyocytes may not be able to fully replicate human VMC, the role of CTRP9 and THBS1 in the VMC process *in vivo* needs further investigation. In addition, this study only shows the interaction between CTRP9 and THBS1, and studying changes in this interaction in clinical samples will bring greater value to VMC treatment.

## Data Availability

The data used to support the findings of this study are available from the corresponding author upon request.

## Supplementary Materials

Supplementary Materials The CTRP9 expression in different age groups of VMC and the VP1 expression in different treated groups of H9c2 cells.
