# Supplementary material for: C1q/TNF-Related Protein 9 Inhibits Coxsackievirus B3-Induced Injury in Cardiomyocytes through NF-κB and TGF-β1/Smad2/3 by Modulating THBS1
Source: Mediators Inflamm. 2020 Dec 19;2020:2540687. doi: 10.1155/2020/2540687 (PMC7769632; doi:10.1155/2020/2540687)
Supplement: Supplementary Materials — The CTRP9 expression in different age groups of VMC and the VP1 expression in different treated groups of H9c2 cells. [file 2540687.f1.docx]

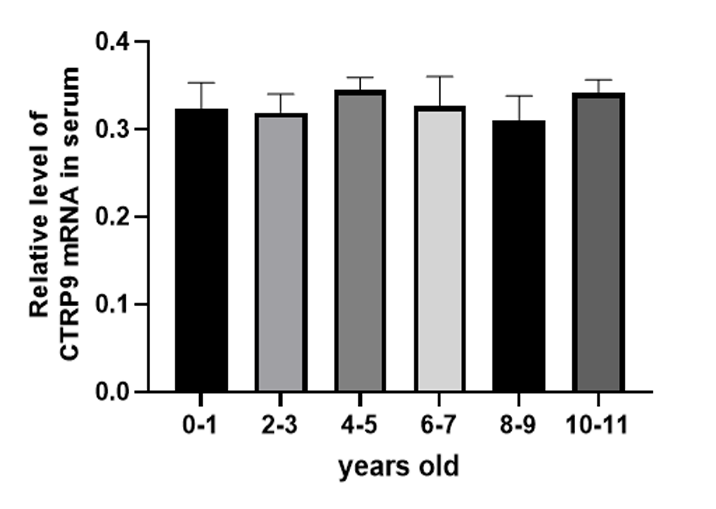


Fig.1 The relative expression of serum CTRP9 mRNA in different age groups of VMC group showed no significant difference.


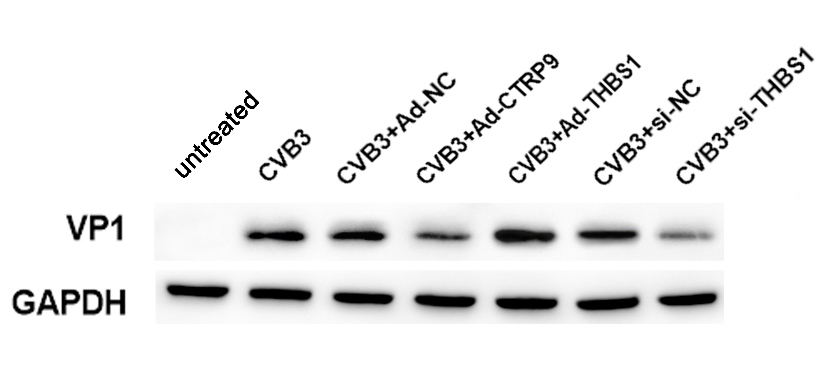


Fig.2 The VP1 expression of H9c2 cells was decreased by Ad-CTRT9 and si-THBS1 while increased by Ad-THBS1. Antibodies for detecting viral VP1 was purchased from Leica Biosystems Newcastle.
